# Prevalence of Cognitive Decline in Type 2 Diabetes Mellitus Patients: A Real-World Cross-Sectional Study in Mysuru, India

**DOI:** 10.3390/jpm13030524

**Published:** 2023-03-15

**Authors:** Nabeel Kinattingal, Seema Mehdi, Krishna Undela, Shahid Ud Din Wani, Mansour Almuqbil, Sultan Alshehri, Faiyaz Shakeel, Mohammad T. Imam, Santhepete N. Manjula

**Affiliations:** 1 Department of Pharmacology, JSS College of Pharmacy, JSS Academy of Higher Education and Research, Mysuru 570015, India; 2 Department of Pharmacy Practice, National Institute of Pharmaceutical Education and Research (NIPER), Guwahati 781101, India; 3 Department of Pharmaceutical Sciences, School of Applied Science and Technology, University of Kashmir, Srinagar 190006, India; 4 Department of Clinical Pharmacy, College of Pharmacy, King Saud University, Riyadh 11451, Saudi Arabia; 5 Department of Pharmaceutical Sciences, College of Pharmacy, AlMaarefa University, Ad Diriyah 13713, Saudi Arabia; 6 Department of Pharmaceutics, College of Pharmacy, King Saud University, Riyadh 11451, Saudi Arabia; 7 Department of Clinical Pharmacy, College of Pharmacy, Prince Sattam Bin Abdulaziz University, Al-Kharj 11942, Saudi Arabia

**Keywords:** cognitive decline, diabetes, mild cognitive impairment, Montreal Cognitive Assessment

## Abstract

The goal of this research is to study the prevalence of cognitive impairment in diabetes mellitus (DM) patients and establish the necessity of detecting and treating it early in these patients. A cross-sectional study was conducted at a tertiary care hospital in Mysuru for 4 months examined diabetic patients (test) and nondiabetic subjects (control) for cognitive decline using the Montreal Cognitive Assessment (MoCA) tool. Cognitive functions such as visuospatial/executive function, naming, attention, language, abstraction, delayed recall, and orientation were assessed in both groups. The diabetic group showed a significantly lower total MoCA score than the non-diabetic group (18.99 ± 0.48 and 26.21 ± 0.46, respectively; *p* < 0.001). Assessment of scores in diabetic patients demonstrated the significant influence of age demographics on cognitive impairment (*p*-value < 0.001). Furthermore, a higher proportion of diabetic patients displayed cognitive impairment despite a higher score in a single subdomain, making it evident that diabetes is diverse and multifactorial in origin, where oxidative stress and inflammatory responses play a predominant role. This study suggested that the local T2DM population residing in Mysuru (India) has a high prevalence of cognitive impairment, evident from poor performance in almost all cognitive domains assessed by MoCA. Future studies could examine the generalizability of cognitive function findings in diabetic patients across diverse geographic regions and ethnic groups, as well as investigate interventions such as lifestyle modifications and medication to prevent or delay cognitive decline in those with diabetes.

## 1. Introduction

A serious issue in terms of world health is diabetes mellitus (DM). The steep surge in DM cases worldwide and the future projections are alarming. In 2019, 463 million people (9.3%) worldwide suffered from DM, which is projected to rise to 578 million (10.2%) by 2030 and 700 million people (10.9%) by 2045 [1]. Further, the International Diabetes Federation revealed that in 2019, India witnessed the second-highest number of individuals suffering from diabetes (77 million; 8.9% prevalence) [2].

DM is a metabolic disorder, and its various complications result in compromised quality of life [3,4]. The risk of peripheral vascular disease, cardiovascular disease, peripheral neuropathy, nephropathy, and retinopathy in DM is well established [5,6]. Researchers have explored the impact of diabetes on the cognitive system and memory disorders [7]. Earlier studies revealed a direct link between DM and cognitive impairment and dementia [8,9]. Additionally, other researchers have linked type 2 diabetes mellitus (T2DM) to changes in memory, psychomotor speed, visuospatial functions, frontal executive functions, processing speed, verbal fluidity, attention, and complex motor abilities [10,11].

Neurons, being metabolically active, depend on brain glucose metabolism for function and survival [12]. Hence, hyperglycemia and insulin resistance (IR), the principal pathological characteristics of T2DM, might result in diabetes-associated cognitive dysfunction [13]. Furthermore, diabetic encephalopathy is another significant risk factor for cognitive dysfunction, dementia, and consequently Alzheimer’s disease. Many studies have illustrated that diabetes increases the risk of cognitive impairment due to impaired insulin signaling, increased oxidative stress, and inflammation [14]. A research study identified structural changes in the brains of diabetic patients, such as hippocampal injury, reduced gray matter density, altered white matter microstructure, and atrophy, indicating a higher risk of neurocognitive dysfunction in these patients [15].

The current management strategies for T2DM do not primarily target cognitive dysfunction. The increasing prevalence of diabetes and the aging population suggest that diabetes-related cognitive decline would substantially affect the globe and the nation, which defines the need to explore the less addressed cognitive decline in diabetes. Further, a recent study conducted in the rural district of South Karnataka established that one individual in every 5^th^ family or one of every 12 individuals above 20 years was diabetic and that the overall prevalence rate of DM was higher than the national prevalence rate in different age groups and family sizes [16]. Hence, to examine the impact of diabetes on cognitive impairment in the local population, a cross-sectional observational study was carried out at JSS Hospital, Mysuru, India.

## 2. Materials and Methods

### 2.1. Methods

After receiving human ethical approval, a cross-sectional study was carried out for 4 months at a tertiary care hospital in Mysuru, India. The participants were grouped into two groups: test and control. This study included body mass index (BMI), smoking, and education score as factors that might influence cognitive function in diabetic individuals. BMI was classified based on WHO criteria, smoking status was classified as current, ex-smoker, or non-smoker, and the education score was based on the number of years of formal education completed, classified as grades. These classifications are supported by guidelines from organizations such as WHO and CDC and previous research on cognitive reserve and educational attainment [17,18]. The Montreal Cognitive Assessment (MoCA), a standardized and rapid tool to assess cognitive abilities, screened the subjects in both groups; in the diabetic group, patients were asked about adherence to regular medication and their postprandial blood sugar (PPBS) [19,20].

The MoCA scale’s assessment of cognitive decline used performance scores from 0–6, 0–5, 0–3, 0–3, 0–7, and 0–6, respectively, in the domains of name, attention, language, abstraction and delayed recall, and orientation. The lowest score of zero signifies poor performance in that domain [21].

### 2.2. Participants

#### Test Group

The T2DM patients (inpatients and outpatients) from the General Medicine Department were screened for eligibility as per the inclusion criteria and included in the test group.

Inclusion criteria for the test group:

- T2DM patients of any gender aged between 30 years and 60 years;
- T2DM patients with minimal literacy (able to read and write) in Kannada or English.

Exclusion criteria for the test group:

- Patients with a history or present condition of type 1 diabetes, secondary diabetes, or gestational diabetes;
- Patients with psychiatric illness or on medication having psychoactive activity;
- Patients suffering from severe diseases such as, but not confined to, cancer (diagnosed less than five years ago) or severe autoimmune diseases;
- Patients with neurological diseases;
- Patients whose caregivers did not give consent.

Subjects in the control group followed the same inclusion and exclusion criteria except that these were individuals without T2DM.

### 2.3. Settings

The subjects were interviewed after obtaining informed consent. Demographic and anthropometric data were collected from their medical records and interviews and recorded in a specially designed data collection form. The subjects with MoCA scores below 26 were considered cognitively impaired, and those above 26 were considered normal.

### 2.4. Study Size

The sample size was calculated using N = 4PQ/d2, and 100 volunteers were chosen for the control group and 100 patients were chosen for the test group. In each group, there were more male patients than females.

### 2.5. Statistical Analysis

This study was conducted in two arms, each consisting of 100 subjects. Data collected from the participants were observed to follow a normal distribution and was subsequently analyzed using SPSS Version 23. Descriptive statistical measures such as the mean with standard deviation were utilized, while inferential statistical tests such as the chi-square test, Student’s t-test, and multivariate correlational analysis were performed to derive relevant insights from the data. The *p*-value was calculated using MedCalc statistical software (Version 20.218), which helped determine the level of statistical significance associated with the results obtained.

## 3. Results

Table 1 lists the demographic and anthropometric information, including age, gender, marital status, height, weight, BMI, level of education, place of residence, and status as a smoker compared by the chi-square test.

The mean age of participants was 51 ± 8 and 50 ± 8 years for the diabetic and nondiabetic groups, respectively. Both groups recruited a higher proportion of male subjects. A larger proportion of individuals were married. Moreover, most of the recruited population showed a normal BMI (49% of patients in the diabetic group and 74% in the nondiabetic group). A larger percentage of subjects had education levels higher than grade 6. In the diabetic group, a larger population belonged to the urban community than in the nondiabetic group, where a larger population belonged to the rural community. Most of the participants in the test and control groups were nonsmokers. A significant difference (*p* < 0.001) was observed for demographic measures such as age group, gender, marital status, mean height, BMI, educational level, area, and smoking status.

Both groups were evaluated for cognitive function. The total MoCA score and the performance score in each cognitive subdomain are summarized in Table 2.

As evident from Table 2, the diabetic group showed a significantly lower total MoCA score than the non-diabetic group (18.99 ± 0.48 and 26.21 ± 0.46, respectively; *p* < 0.001). However, when analyzed individually, the test group showed statistically lower cognitive function in terms of visuospatial/executive function, naming, attention, abstraction, and delayed recall but similar functions in language and orientation to the control group.

The mean MoCA score for the diabetic group concerning the patient demographics was evaluated further, as depicted in Table 3.

The diabetic patients showed no significant difference in their mean MoCA scores based on their demographic factors except their age. The patients displayed a higher MoCA score and better cognition at a younger age, which decreased in elder patients. Moreover, the patients with a lesser duration of diabetes mellitus and the patients taking their medications regularly scored a little higher than those with a longer duration of diabetes mellitus or who were not regular in taking their medication. However, the difference in scores was statistically insignificant.

A relationship between the MoCA score and PPBS among the test group was determined to correlate the impact of hyperglycemia on cognitive dysfunction. The data are presented in Table 4.

In this study, PPBS levels were recorded for participants in the test group. Analysis of the data revealed that 40% of the participants had PPBS levels above 250 mg/dL, 16% maintained PPBS levels between 75 and 150 mg/dL, and the remaining participants had PPBS levels in the range of 151–200 mg/dL (25%), and 201–250 mg/dL (19%).

The results further indicated that diabetic patients who exhibited mild cognitive impairment (MCI) had an average PPBS level of 249.85 (107.973) mg/dL, whereas those with normal cognitive function (8%) demonstrated an average PPBS level of 229.75 (85.081) mg/dL, although this difference was not statistically significant.

**Figure 1:**
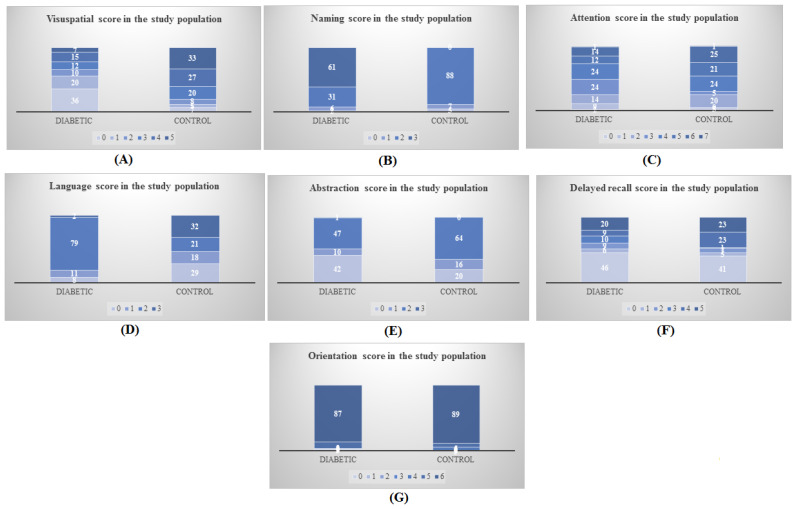
Subdomain scores in study subjects: (**A**) visuospatial score, (**B**) naming score, (**C**) attention score, (**D**) language score, (**E**) abstraction score, (**F**) delayed recall score, and (**G**) orientation score.

The results of this study’s last stage compared the MoCA scores of the two groups in order to examine various cognitive subdomains, such as visuospatial/executive function, naming, attention, language, abstraction, delayed recall, and orientation. The data obtained are presented in Table 5 and Figure 1A–F.

The results show that in the diabetic group, a large proportion of the patients (71.42%) with a high visuospatial/executive function score were cognitively impaired. In contrast, in the nondiabetic group, all the individuals with high visuospatial/executive function scores (4 and 5) showed normal cognition. Similarly, for the naming subdomain, 88.52% of diabetic patients showed cognitive impairment despite a maximum score of 3, while in the non-diabetic group, only 30.76% of individuals with a score of 3 showed cognitive impairment, which was significant (*p* < 0.0001). Similar results were obtained for attention, i.e., 71.42% of patients showed cognitive impairment despite a high score of 6, compared to 43.47% of non-diabetic individuals with a high score of 6. For the language subdomain, only 50% of the diabetic patients with a maximum score of 3 showed normal cognition, compared to 93.75% of non-diabetic individuals with a maximum score of 3. For the abstraction subdomain, 12.76% of the diabetic group and 86.46% of non-diabetic individuals showed normal cognition for a score of 2 which was significant (*p* < 0.0001). For delayed recall of the diabetic patients with a maximum score of 5, only 25% showed normal cognition against 75% of individuals in the nondiabetic group. Finally, for the orientation subdomain of the diabetic patients scoring a maximum score of 6, only 9.19% showed normal cognition compared to 74.41% of individuals in the nondiabetic group, which was significant (*p* < 0.0001).

**Figure 2:**
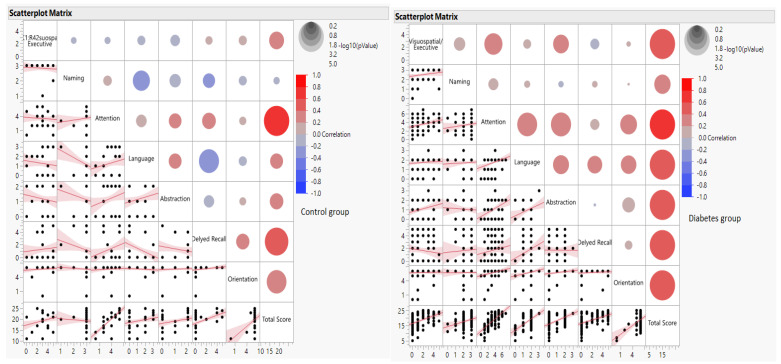
Scatter plot and bubble distribution of multivariate correlation analysis.

A multivariate correlation analysis was performed to determine the association between the total score (<25), indicating cognitive impairment, and the subdomain scores. Table 6 reveals a significant (*p* < 0.01) moderate-to-strong correlation (r = 0.4 − 0.7) between the total and the sub-domain scores in the diabetes group, indicating that the diabetic subjects with cognitive impairment (MOCA score < 25) also had poor subdomain scores. In contrast, the control group did not depict any relation between a subdomain and total cognitive scores. The scatter plot and bubble plot in Figure 2 show the degree of correlation between the various subdomains and total scores.

## 4. Discussion

The MoCA is a widely accepted tool for assessing MCI and is primarily utilized to detect potential cognitive decline in patients [22]. In this study, we utilized the MoCA scale to assess cognitive function in individuals with diabetes, encompassing their comprehensive cognitive abilities and performance across various cognitive domains. Additionally, we included a control group of nondiabetic individuals to facilitate intergroup comparison. The utilization of this standardized tool ensured consistent and objective assessment of cognitive performance, enabling us to identify potential cognitive impairment in diabetic individuals and contrast it with the cognitive function of non-diabetic individuals.

Normal cognitive abilities are defined by a MoCA score of 26 or higher. In this study, the non-diabetic (control) group exhibited a mean MoCA score of >26 (26.21 ± 0.46), while the diabetic group demonstrated a low MoCA score of 18.99 ± 0.48, indicating a significant association between diabetes and cognitive impairment. These findings are consistent with previous studies reporting a higher incidence of mild MCI in patients with T2DM. Specifically, sociodemographic factors were associated with cognitive impairment in 50% of the diabetic study population, and another study demonstrated statistically significant MCI in T2DM patients with higher levels of HbA1C, FBS, and PPBS levels [23,24].

The control group demonstrated significantly superior performance compared to the test group across various subdomains of the MoCA tool, including visuospatial/executive function, naming, attention, abstraction, and delayed recall. However, both groups had average scores in the language and orientation domains, indicating that DM may not cause problems with language skills such as comprehension, fluency, or grammar or with orientation skills such as the capacity to judge time and place [25].

Furthermore, an assessment of the influence of demographics on cognitive impairment revealed that age was the only factor that statistically influenced cognitive impairment (*p*-value < 0.001). These results agreed with a recent study illustrating the age-related prevalence of MCI in T2DM patients in the Indian population, which demonstrated a consistently lower performance on the MoCA scale with increasing age groups of the diabetic population [26]. Age-related cognitive decline is a well-documented phenomenon and may be attributed to reduced hippocampal size, leading to compromised neuroplasticity and neurodegeneration [27].

This study determined that various factors such as gender, education level, geographic location, smoking habits, PPBS, duration of diabetes, and regular medication use did not significantly impact the cognitive performance of diabetic individuals on the MoCA scale. The majority (61%) of diabetic participants had a diabetes duration of at least 5 years, while only a small percentage (13%) had diabetes for 10 or more years. Furthermore, over 93% of participants were taking regular medication for diabetes, with oral anti-diabetic medication being the most common treatment; however, medication use did not significantly affect MoCA scores. Notably, the participant with the highest MoCA score of 23(0) was both single and married, while the lowest score of 17.43 (5.224) was recorded in seven participants who did not adhere to their medication regimen consistently.

Analysis of PPBS data in the test group revealed that 40% of the participants had their PPBS above 250 mg/dL while 19% were in the range of 201–205 mg/dL, 25% had PPBS in the range of 151–200, and 16% were within the range of 75–150 mg/dL. The diabetic patients assessed with MCI (92%) had a PPBS of 249.85 (107.973), and those who had normal cognition (8%) were found to have 229.75 (85.081) mg/dL, but the data were not significant.

This study revealed that a significant number of individuals with diabetes showed mild MCI despite scoring well in specific cognitive domains. Conversely, individuals without diabetes who scored highly in the same cognitive domains demonstrated normal cognitive function. The findings suggest that the prevalence of MCI among diabetic individuals may be because of the complex and diverse nature of diabetes, which has multiple contributing factors, including oxidative stress and inflammatory responses. These factors might play a crucial role in the development of cognitive impairment in diabetic individuals, as supported by previous research [28,29,30].

## 5. Conclusions

According to the observational cross-sectional study, there is a substantial prevalence of cognitive impairment among the local T2DM community in Mysuru, India, as evidenced by their subpar performance on practically every cognitive domain tested by MoCA. This study had the limitation that it was a single-center study with a limited population size. Moreover, due to the unavailability of HbA1C data for the subjects, this study could not infer the association between cognitive decline and HbA1C, an essential marker of diabetes. Additionally, the lack of characterization of diabetic complications cannot be substituted by disease onset. However, the higher occurrence of MCI in the diabetic group suggested that detecting MCI in T2DM should be incorporated into routine clinical practice and supported with early treatment through lifestyle and pharmacological interventions. Further studies are required to explore the therapeutic use of anti-glycemic agents and antioxidants to combat the hyperglycemia, oxidative stress, and inflammatory conditions existing in the diabetic brain and alleviate the cognitive decline across diverse geographic regions and ethnic groups, as well as investigate interventions such as lifestyle modifications and medication to prevent or delay cognitive decline in T2DM patients.

## Figures and Tables

**Table 1:** Sociodemographics of participants in the test and control groups.

Demographics	Test Group (Diabetics)	Control Group (Nondiabetics)	*p*-Value
Mean Age	51 ± 9	50 ± 8	<0.09
Age group			<0.001
30–40 years	18	54	
41–50 years	25	39	
51–60 years	57	07	
Gender			<0.001
Male	53	77	
Female	47	23	
Marital Status			<0.001
Married	99	84	
Unmarried	1	16	
Mean Weight (kg)	66.98 ± 11.47	66.20 ± 11.32	0.708
Mean Height (cm)	162.57 ± 7.95	167.91 ± 8.24	<0.001
BMI			<0.001
Normal	49	70	
Underweight	04	07	
Overweight	36	09	
Obese	11	14	
Education			<0.001
Grade 5 or below	28	00	
Grade 6 or above	72	100	
Area			<0.001
Rural	65	36	
Urban	35	64	
Smoking			<0.001
Current smokers	5	27	
Ex-smokers	7	5	
Nonsmokers	88	68	

**Table 2:** Mean MoCA score in the test and control groups for total cognitive function and each subdomain.

Cognitive Domain	Highest Score	Test Group (Diabetic) Mean Score (SD)	Control Group (Nondiabetic) Mean Score (SD)	*p*-Value
Total Score on MoCA	30	18.99 (0.48)	26.21 (0.46)	<0.001
Visuospatial/Executive Function	5	1.71 (0.17)	4.02 (0.16)	<0.001
Naming	3	2.51 (0.07)	2.89 (0.05)	0.001
Attention	6	3.57 (0.16)	5.21 (0.17)	<0.001
Language	3	1.75 (0.06)	1.75 (0.17)	1.000
Abstraction	2	1.07 (0.10)	1.68 (0.11)	<0.001
Delayed Recall	5	1.9 (0.20)	4.07 (0.20)	<0.001
Orientation	6	5.72 (0.10)	5.86 (0.14)	0.414

**Table 3:** MoCA score as per the demographics of the test group.

Demographics	N = 100	Mean MoCA Score (SD)	*p*-Value
Gender			
Male	53	19.23 (4.84)	0.602
Female	47	18.72 (4.74)	
Age			
30–40	18	22.33 (3.11)	<0.001
41–50	25	19.96 (4.68)	
51–60	57	17.51 (4.67)	
Marital Status			
Married	1	23.00 (0.0)	0.402
Unmarried	99	18.95 (4.79)	
Education			
Less or equal to 5 years of formal education	28	17.71 (5.50)	0.096
More or equal to 6 years of formal education	72	19.49 (4.41)	
Area			
Rural	65	18.69 (4.76)	0.398
Urban	35	19.54 (4.84)	
Smoking			
Current	5	18.40 (4.45)	0.957
Ex	7	18.86 (4.67)	
Non	88	19.03 (4.85)	
DM Years			
0–5	61	19.57 (4.59)	0.302
6–10	26	17.92 (5.59)	
Above 10	13	18.38 (3.64)	
PPBS			
75–150	16 (16)	17.75 (5.323)	<0.553
151–200	25 (25)	19.04 (3.942)	
201–250	19 (19)	20.11 (4.999)	
Above 250	40 (40)	18.93 (4.969)	
Regular Medication			
Yes	93	19.11 (4.75)	0.373
No	7	17.43 (5.22)	

**Table 4:** Correlation of the MoCA score with PPBS in the test group.

MoCA Score	No. of Patients	PPBS Mean (SD)	*p* Value
0–25	92	249.85 (107.97)	<0.601
Above 26	8	229.75 (85.08)	

**Table 5:** The proportion of participants showing cognitive impairment or normal cognition concerning their scores in each cognitive subdomain.

Cognitive Domain	Score	Test			Control			*p*
		No of Patients Total	Cognitive Impairment n (%)	Normal Cognition n (%)	No of Patients Total	Cognitive Impairment n (%)	Normal Cognition n (%)	
Visuospatial/Executive Function	0	36	36 (100)	0 (0)	1	1 (100)	0 (0)	
	1	20	19 (95)	1 (5)	0	0 (0)	0 (0)	0.5366
	2	10	10 (100)	0 (0)	2	2 (100)	0 (0)	0.1657
	3	12	11 (91.66)	1 (8.33)	9	9 (100)	0 (0)	
	4	15	11 (73.33)	4 (26.66)	14	0 (0)	14 (100)	0.3175
	5	7	5 (71.42)	2 (28.57)	18	0 (0)	18 (100)	0.0212
Naming	0	2	2 (100)	0 (0)	0	0 (0)	0 (0)	0.6209
	1	6	6 (100)	0 (0)	0	0 (0)	0 (0)	0.5381
	2	31	30 (96.77)	1 (3.22)	5	0 (0)	5 (100)	0.0720
	3	61	54 (88.52)	7 (11.47)	39	12 (30.76)	27 (69.23)	<0.0001
Attention	0	1	1 (100)	0 (0)	1	1 (100)	0 (0)	
	1	10	10 (100)	0 (0)	0	0 (0)	0 (0)	0.5188
	2	14	14 (100)	0 (0)	0	0 (0)	0 (0)	0.5102
	3	24	24 (100)	0 (0)	0	0 (0)	0 (0)	0.5012
	4	24	21 (87.5)	3 (12.5)	9	1 (11.11)	8 (88.88)	0.0291
	5	12	11 (91.66)	1 (8.33)	11	0 (0)	11 (100)	0.0277
	6	14	10 (71.42)	4 (28.57)	23	10 (43.47)	13 (56.52)	0.0888
	7	1	1 (100)	0 (0)	1	0 (0)	0 (0)	0.7074
Language	0	8	8 (100)	0 (0)	8	8 (100)	0 (0)	
	1	11	11 (100)	0 (0)	11	1 (9.09)	10 (90.90)	0.0119
	2	79	72 (91.13)	7 (8.86)	9	2 (22.22)	7 (77.77)	0.0239
	3	2	1 (50)	1 (50)	16	1 (100)	15 (93.75)	0.0829
Abstraction	0	42	41 (97.61)	1 (2.38)	7	7 (100)	0 (0)	0.3173
	1	10	9 (90)	1 (10)	0	0 (0)	0 (0)	0.5874
	2	47	41 (87.23)	6 (12.76)	37	5 (13.51)	32 (86.46)	<0.0001
	3	1	1 (100)	0 (0)	7	7 (100)	0 (0)	
Delayed Recall	0	46	46 (100)	0 (0)	3	3 (100)	0 (0)	
	1	6	6 (100)	0 (0)	0	0 (0)	0 (0)	0.5381
	2	9	8 (88.88)	1 (11.11)	2	0 (0)	2 (100)	0.2093
	3	10	9 (90)	1 (10)	1	0 (0)	1 (100)	0.3137
	4	9	8 (88.88)	1 (11.11)	18	4 (22.22)	14 (77.77)	0.0024
	5	20	15 (75)	5 (25)	20	5 (25)	15 (75)	0.0071
Orientation	0	2	2 (100)	0 (0)	1	1 (100)	0 (0)	
	1	0	0 (0)	0 (0)	0	0 (0)	0 (0)	
	2	1	1 (100)	0 (0)	0	0 (0)	0 (0)	0.7074
	3	1	1 (100)	0 (0)	0	0 (0)	0 (0)	0.7074
	4	0	0 (0)	0 (0)	0	0 (0)	0 (0)	
	5	9	9 (100)	0 (0)	0	0 (0)	0 (0)	0.5221
	6	87	79 (90.80)	8 (9.19)	43	11 (25.58)	32 (74.41)	<0.0001

**Table 6:** Multivariate correlation analysis.

	Correlation with Total MOCA Score, r		Correlation Probability (*p*-Value)	
	Control	Diabetes	Control	Diabetes
Visuospatial/Executive	0.27	0.47	0.02	<0.0001
Naming	−0.07	0.26	0.55	0.01
Attention	0.79	0.71	<0.0001	<0.0001
Language	0.23	0.53	0.06	<0.0001
Abstraction	0.24	0.51	0.04	<0.0001
Delayed Recall	0.47	0.5	<0.01	<0.0001
Orientation	0.39	0.45	0.001	<0.0001
Total Score	1	1	<0.0001	<0.0001

## Data Availability

The data presented in this study are available on request from the corresponding author.
